# Construction and validation of a nomogram model to predict the overall survival rate of esophageal cancer patients receiving neoadjuvant chemotherapy: A population-based study

**DOI:** 10.3389/fsurg.2022.1066092

**Published:** 2023-01-06

**Authors:** Ying Yang, Changjin He

**Affiliations:** Department of Thoracic Surgery, Ningde Municipal Hospital of Ningde Normal University, Ningde, China

**Keywords:** neoadjuvant chemotherapy, esophageal cancer, nomogram model, overall survival, clinical research

## Abstract

**Introduction:**

The development of neoadjuvant chemotherapy(nCT) improves the overall survival (OS) of patients with esophageal cancer(EC). The aim of this study was to determine the independent prognostic factors of EC patients receiving nCT, and to construct a nomogram model for predicting OS.

**Method:**

This retrospective analysis was conducted from the National Cancer Institute's Surveillance Epidemiology and End Results, Clinicopathological data of patients with EC who received nCT from 2004 to 2015. The included patients were randomly divided into the training cohort and the validation cohort. Univariate and multivariate Cox proportional hazards models were used to analyze the patients in the training cohort to determine the independent prognostic factors. Based on the independent prognostic variables, nomogram models for 1-year, 2-year and 3-year OS were constructed. The receiver operating characteristic (ROC) and area under curve (AUC) were used to evaluate the discriminative ability. The calibration curves, decision curve analysis (DCA) and Kaplan-Meier (K-M) survival analysis were used to evaluate the predictive accuracy and clinical application value.

**Results:**

A total of 2,493 patients were enrolled, with 1,748 patients in the training cohort and 745 patients in the validation cohort. Gender, marital status, tumor pathological grade, T stage, N stage, and M stage were identified as independent prognostic factor (*P* < 0.05). A novel nomogram model was constructed. ROC curve analysis revealed that the model had moderate predictive performance, which was better than that of the AJCC TNM staging system.The calibration curves showed a high agreement between the actual observed values and the predicted values. The DCA suggested that the newly constructed prediction model had good clinical application value. K-M survival analysis showed that the model was helpful to accurately distinguish the prognosis of patients with different risk levels.

**Conclusions:**

Gender, tumor pathological grade, marital status, T stage, N stage and M stage were identified as independent prognostic factors for overall survival of patients with esophageal cancer who received neoadjuvant chemotherapy. A nomogram prediction model was established, which was helpful to accurately and reliably predict the overall survival rate of patients with esophageal cancer who received neoadjuvant chemotherapy at 1, 2 and 3 years.

## Introduction

Esophageal cancer (EC) is a common gastrointestinal malignancy, ranking the 7th among the most common cancers in the world, and the 6th among cancer-related deaths (1, 2). Esophageal squamous cell carcinoma (ESCC) and esophageal adenocarcinoma (EAC) are the two main pathological types of EC.The detection rate and accuracy of imaging examinations for EC are limited due to the occult early symptoms and relatively limited lesion scope.At the same time, the EC clinical tumor markers (cytokeratin 19 fragment, squamous cell carcinoma antigen and carcinoembryonic antigen) in the detection and lack of ideal sensitivity and specific degrees (3, 4). Most patients with EC are at an locally advanced stage at the time of initial diagnosis, and the five-year survival rate after esophageal surgery alone is less than 25% (5).

In recent years, the multidisciplinary combination of neoadjuvant therapy has been continuously discussed in the clinical management of patients with EC, among which neoadjuvant chemotherapy(nCT) has been recommended as the first-line treatment option for locally advanced EC by NCCN guidelines (6). The purpose of nCT is to reduce the tumor lesion, reduce the pathological stage, improve the surgical resection rate and thus help to prolong the long-term survival. At present, the commonly used nCT is platinum combined with fluorouracil or paclitaxel (7). Ando N et al. reported that the nCT regimen of cisplatin plus fluorouracil could prolong the disease-free survival of EC patients (8). In addition to initiating neoadjuvant chemotherapy, determining prognostic factors and prognostic assessment are also important components of clinical management of EC patients. The TNM staging system proposed by the American Joint Committee on Cancer (AJCC) has been regarded by clinicians as the main basis for disease progression and prognosis evaluation of cancer patients. The primary tumor stage(T), lymph node involvement(N) and distant organ metastasis (M) are the three dimensions to evaluate the tumor stage. Although the AJCC staging system has been widely used, and its prognostic value and role in tumor patient stratification have been consistently confirmed in clinical practice. However, recent studies have consistently found that in addition to AJCC staging system, other clinical factors are also significantly associated with the prognosis of esophageal cancer patients. Qian et al. found that in addition to AJCC stage, patients' age, gender, race, and tumor grade were independently related to the prognosis of esophageal adenosquamous carcinoma (9). In addition, Huang et al. conducted prognostic analysis and constructed a survival prediction model for osteosarcoma patients who received nCT (10). Unfortunately, there was still limited studies focusing on constructing a nomogram model to predict the survival of EC patients receiving nCT.

Therefore, this study aimed to determine the independent prognostic factors for overall survival(OS), and to establish a nomogram model for predicting the 1-year, 2-year and 3-year OS of EC patients who received nCT. The representative cohort was from the Surveillance, Epidemiology and End Results (SEER) database from 2004 to 2015.

## Methods

### Study design

This study utilizes SEER*Stat version 8.3.9 (https://SEer.cancer.gov/) access the SEER database (covering 18 registries) established by the National cancer Institute of the United States. This publicly available database records the clinical data, pathological data and follow-up information of a large number of patients with malignant tumors in the United States, which is an important tool for the study of cancer epidemiology and prognosis of cancer patients. We retrospectively collected basic demographic information, clinicopathological data, treatment information, survival status and follow-up data of patients diagnosed with EC from 2004 to 2015 in the SEER database. Patients diagnosed with EC were staged according to the American Joint Committee on Cancer (AJCC) TNM staging system. Considering thta SEER database does not publish personally identifiable information of patients, the analysis of data in this study was exempt from medical ethical review, and informed consent was not required. All procedures performed in studies involving human participants comply with the 1,964 Declaration of Helsinki and its subsequent amendments or similar ethical standards.

### Inclusions and exclusions

The inclusion criteria were as follows: (1) Patients with histologically diagnosed EC between 2004 and 2015; (2) Patients whose primary site of malignant tumor was esophagus (tumor location coded C15.0-C15.9); (3) Patients with EC as primary tumor; (4) patients receiving nCT. The exclusion criteria were as follows: (1) Patients who died during follow-up but whose cause of death was unknown; (2) patients with unknown demographic information; (3) patients with missing or unknown clinicopathological data, including the specific primary location of the tumor, pathological grade of the tumor, AJCC TNM stage of the tumor and tumor size information; (4) Patients with unknown treatment information, including primary site surgery, radiotherapy and chemotherapy.

### Variable extraction and definition

Based on patient-specific information from the SEER database, 13 study variables were extracted for further analysis, including age, sex, race, marital status, primary tumor location, pathological differentiation grade, tumor size, T stage, N stage, M stage, radiation and chemotherapy information.

The primary tumor site was defined according to the International Classification of Neoplastic Diseases (ICD-O) anatomic code. (ICD-O) Codes: Upper third (C15.3), middle third (C15.4), lower third (C15.5) and other sites. Regarding marital status, we excluded misleading data on unmarried or cohabiting couples, and then included “unmarried,” “separated,” “single,” and “widowed” all in the unmarried group. Race includes white, black or other races. To facilitate data processing, patients were divided into three age groups: ≤60 years old and >60 years old. The tumor size was divided into three groups: <5 cm, 5–10 cm, and >10 cm. Overall survival (OS), defined as the interval from the date of diagnosis to the last follow-up or death from any cause, was selected as the primary outcome of this study.

### Statistical analysis

Firstly, all included patients were randomly divided into training cohort and validation cohort according to the ratio of 7:3. Chi-square test and Fisher's exact test or independent sample t-test were used to compare the differences between groups. In the prognostic analysis, the univariate Cox proportional hazards regression model was used to determine the prognostic factors of esophageal cancer patients receiving neoadjuvant chemotherapy, and the statistically significant variables in the univariate Cox proportional hazards regression model analysis (*P* < 0.05) were further included in the multivariate analysis. Variables that remained statistically significant in multivariate Cox proportional hazards regression models were identified as independent prognostic factors for OS in patients with esophageal cancer who received neoadjuvant chemotherapy. Subsequently, we constructed a novel nomogram to predict OS in patients with esophageal cancer receiving neoadjuvant chemotherapy using the “rms” and “regplot” packages, respectively, using identified independent prognostic factors. The differentiation, calibration and clinical value of nomogram were evaluated by multi-dimensional index. The sensitivity and specificity of the model were evaluated by Receiver operator characteristic (ROC) curve and Area under curve (AUC). A calibration curve and 1,000 Bootstrap resampling were used to visually compare the survival probabilities predicted by the nomogram with the actual survival conditions, thus internally and externally evaluating the agreement between the predicted and actual probabilities. Decision analysis curve (DCA) was used to analyze the clinical practicability of the model. Finally, all patients were divided into three risk subgroups: high, medium, and low, according to the optimal cut-off value of the total score determined by X-Tile software. Kaplan-Meier survival analysis and log-rank test were used to compare the survival differences among subgroups.

In this study, all statistical tests were two-sided and *P* < 0.05 was considered statistically significant. Statistical analysis of this study was conducted in IBM SPSS Statistics 25.0. The nomogram construction and validation are carried out in R software (version: 3.6.1).

## Results

### Baseline characteristics

A total of 2,493 eligible patients with EC who received nCT were enrolled in this tudy according to a rigorous screening procedure with inclusion and exclusion criteria. According to the ratio of 7:3, all patients were divided into the training cohort and the validation cohort, of which 1,748 patients were assigned to the training cohort and 745 patients were assigned to the validation cohort. Among inluded patients, 2,122 cases (85.11%) were male and 371 cases (14.88%) were female, and the racial distribution was predominantly white (2,265 cases, 90.85%). There were 1,198 cases (48.05%) with tumor size less than 5 cm, 674 cases (27.04%) with T1–2 stage, 844 cases (33.85%) with N0 stage, and 2,235 cases (89.65%) with M0 stage. Most of the patients were married (70.60%). The detailed basic information of EC patients receiving nCT is summarized in Table 1.

**Table 1:** The demographic and clinicopathological information of esophageal cancer patients receiving neoadjuvant chemotherapy.

Variables		Total cohort (*n*, %)	Training cohort (*n*, %)	Validation cohort (*n*, %)	*p*
		*n* = 2493	*n* = 1748	*n* = 745	
Age	≤60 years	1,068 (42.84)	755 (43.19)	313 (42.01)	0.62
	>60 years	1,425 (57.16)	993 (56.81)	432 (57.99)	
Marital status	Married	1,760 (70.60)	1,236 (70.71)	524 (70.34)	0.89
	Unmarried	733 (29.40)	512 (29.29)	221 (29.66)	
	Black	129 (5.17)	88 (5.03)	41 (5.50)	0.41
Race	Other	99 (3.97)	64 (3.66)	35 (4.70)	
	White	2,265 (90.85)	1,596 (91.30)	669 (89.80)	
Sex	Female	371 (14.88)	261 (14.93)	110 (14.77)	0.96
	Male	2,122 (85.12)	1,487 (85.07)	635 (85.23)	
Primary site	Lower third	2,011 (80.67)	1,430 (81.81)	581 (77.99)	0.10
	Middle third	245 (9.83)	164 (9.38)	81 (10.87)	
	Upper third	25 (1.00)	14 (0.80)	11 (1.48)	
	Other	212 (8.50)	140 (8.01)	72 (9.66)	
Histology	Adenocarcinoma	1,800 (72.20)	1,284 (73.46)	516 (69.26)	0.09
	SCC	437 (17.53)	296 (16.93)	141 (18.93)	
	Other	256 (10.27)	168 (9.61)	88 (11.81)	
Grade	Grade I	122 (4.89)	85 (4.86)	37 (4.97)	0.64
	Grade II	1,074 (43.08)	764 (43.71)	310 (41.61)	
	Grade III	1,265 (50.74)	879 (50.29)	386 (51.81)	
	Grade IV	32 (1.28)	20 (1.14)	12 (1.61)	
T stage	T1–2	674 (27.04)	473 (27.06)	201 (26.98)	1.00
	T3–4	1,819 (72.96)	1,275 (72.94)	544 (73.02)	
N stage	N0	844 (33.85)	593 (33.92)	251 (33.69)	0.95
	N1	1,649 (66.15)	1,155 (66.08)	494 (66.31)	
M stage	_M0	2,235 (89.65)	1,563 (89.42)	672 (90.20)	0.61
	_M1	258 (10.35)	185 (10.58)	73 (9.80)	
Radiation	No	191 (7.66)	135 (7.72)	56 (7.52)	0.92
	Yes	2,302 (92.34)	1,613 (92.28)	689 (92.48)	
Chemotherapy	Without post	2,228 (89.37)	1,559 (89.19)	669 (89.80)	0.70
	With post	265 (10.63)	189 (10.81)	76 (10.20)	
Tumor size	<5 cm	1,327 (53.23)	901 (51.54)	426 (57.18)	0.01
	5–10 cm	1,095 (43.92)	801 (45.82)	294 (39.46)	
	>10 cm	71 (2.85)	46 (2.63)	25 (3.36)	

### Determination of independent prognostic factors of OS

In this study, univariate Cox proportional hazards regression model analysis showed that gender, tumor pathological grade, T stage, N stage, M stage, marital status, and primary tumor site were significantly correlated with OS of EC patients receiving nCT (*P* < 0.05). The above variables were further included in multivariate Cox proportional hazards regression model analysis, and the results of multivariate analysis indicated that gender, tumor pathological grade, T stage, N stage, M stage and marital status were independent prognostic factors for OS in EC patients receiving nCT (Table 2).

**Table 2:** Univariate and multivariate cox analysis of overall survival in EC patients with neoadjuvant chemotherapy.

Variables	Univariate analysis		Multivariate analysis	
	HR (95%CI)	*p*-value	HR (95%CI)	*p*-value
Age				
≤60	Reference			
>60	1.01 (0.9–1.14)	0.83		
Race				
Black	Reference			
Other	0.99 (0.67–1.47)	0.97		
White	0.9 (0.69–1.16)	0.40		
Sex				
Female	Reference		Reference	
Male	1.29 (1.09–1.53)	0.003	1.35 (1.14–1.61)	<0.001
Marital status				
Married	Reference		Reference	
Unmarried	1.16 (1.02–1.31)	0.02	1.2 (1.06–1.36)	0.004
T stage				
T1–2	Reference		Reference	
T3–4	1.31 (1.15–1.5)	<0.001	1.22 (1.06–1.39)	0.005
N stage				
N0	Reference		Reference	
N1	1.47 (1.3–1.67)	<0.001	1.42 (1.25–1.61)	<0.001
M stage				
M0	Reference		Reference	
M1	1.48 (1.24–1.76)	<0.001	1.44 (1.21–1.71)	<0.001
Tumor size				
<5 cm	Reference			
5–10 cm	1.01 (0.9–1.13)	0.89		
>10 cm	1.01 (0.7–1.45)	0.96		
Primary site				
Lower third	Reference			
Middle third	1.03 (0.85–1.26)	0.76	1.21 (0.99–1.48)	0.0673
Upper third	0.88 (0.44–1.77)	0.72	0.8 (0.4–1.62)	0.5423
Other	1.38 (1.13–1.69)	0.002	1.37 (1.12–1.68)	0.0523
Histology				
Adenocarcinoma	Reference			
SCC	0.96 (0.82–1.12)	0.60		
Other	1.17 (0.97–1.42)	0.10		
Grade				
Grade I	Reference		Reference	
Grade II	1.27 (0.95–1.7)	0.11	1.2 (0.9–1.62)	0.22
Grade III	1.54 (1.15–2.05)	0.004	1.46 (1.09–1.95)	0.01
Grade_Grade IV	1.83 (1.01–3.32)	0.045	1.73 (0.96–3.14)	0.07
Radiotherapy				
No	Reference			
Yes	1.04 (0.84–1.28)	0.74		
Chemotherapy				
Withoutpost	Reference			
Withpost	1.01 (0.85–1.21)	0.89		

### Consrtuction and validation of a nomogram model

**Figure 1:**
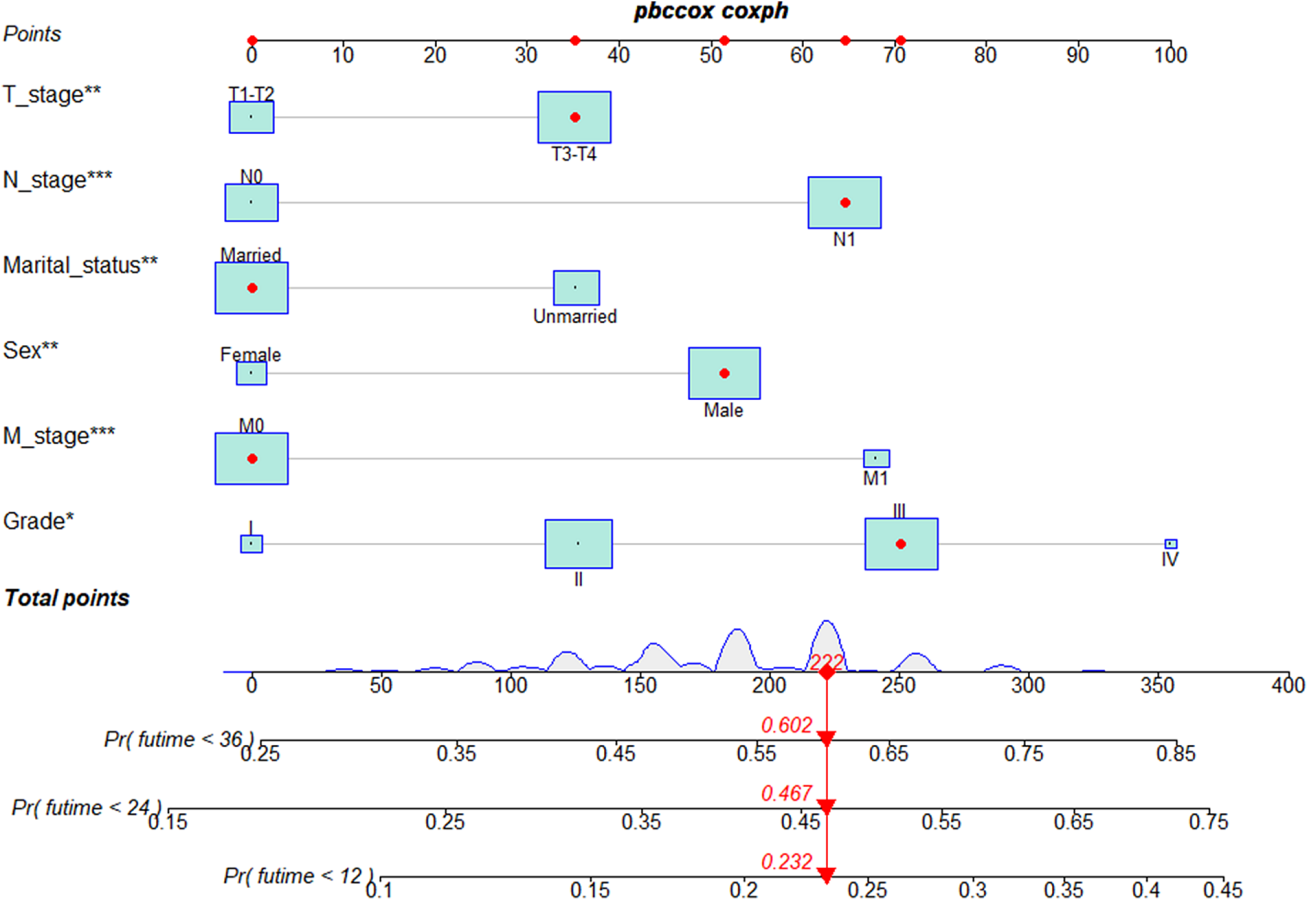
Nomogram predictive models for 1 -, 2 -, and 3-year overall survival in patients with esophageal cancer receiving neoadjuvant chemotherapy.

**Figure 2:**
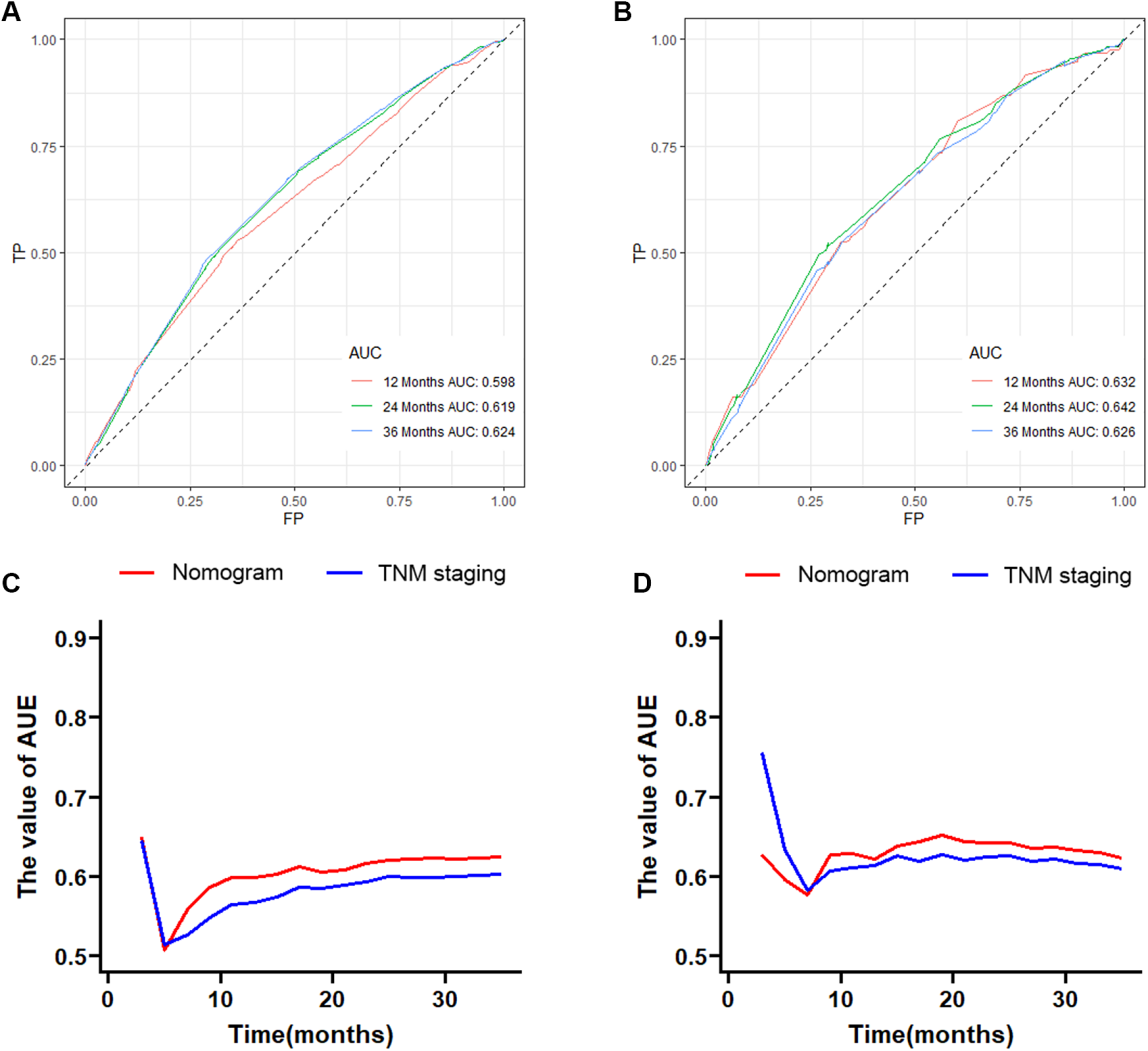
The 1-year, 2-year and 3-year ROC curves and area under the curve of the nomogram prediction model for predicting overall survival of esophageal cancer patients receiving neoadjuvant chemotherapy in the training cohort (**A**) and validation cohort (**B**); the time-dependent ROC curves in the training cohort (**C**) and validation cohort (**D**) were compared between the nomogram prediction model and traditional AJCC TNM staging.

**Figure 3:**
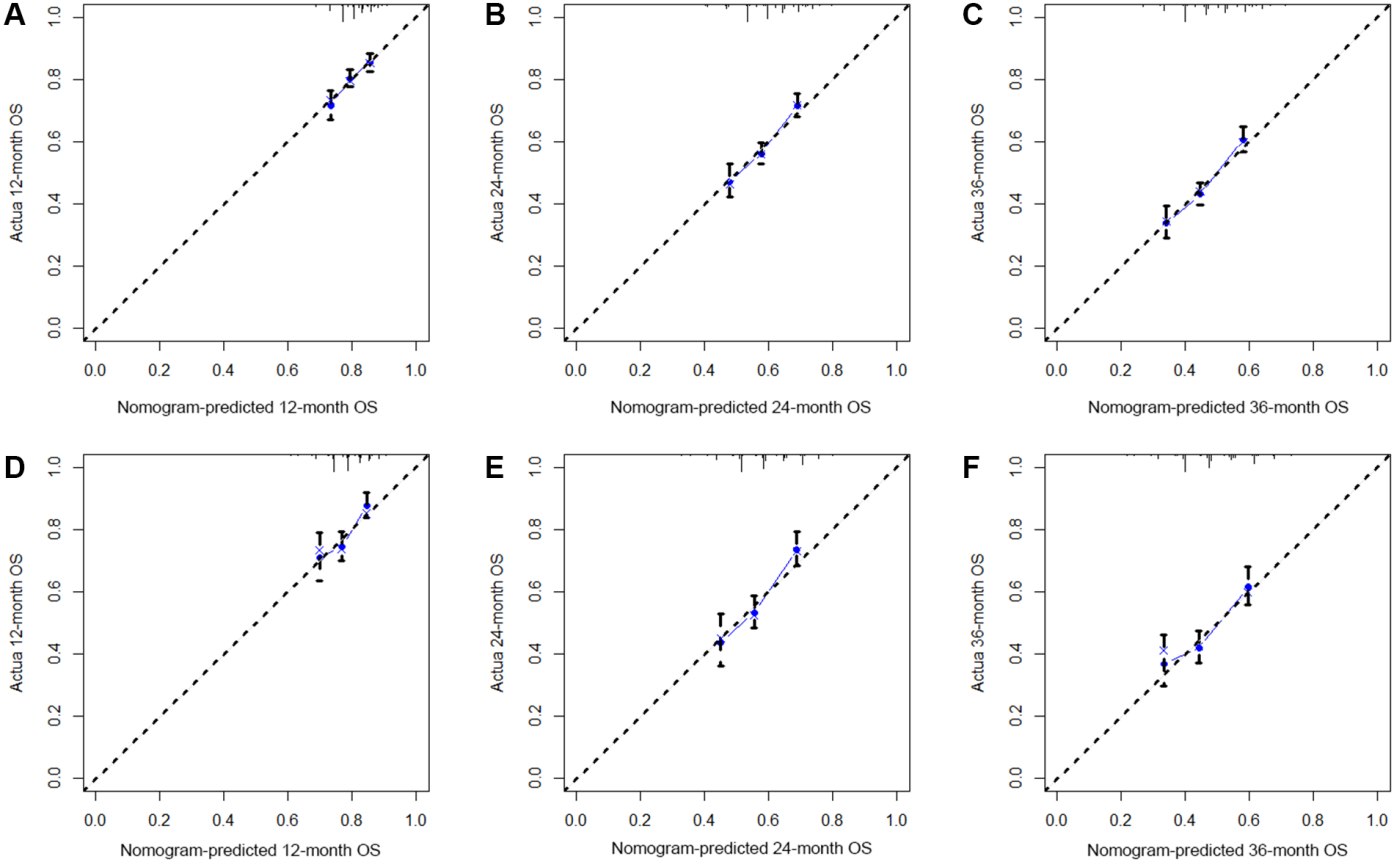
The 1-year (**A**), 2-year (**B**) and 3-year (**C**) calibration curves of the nomogram prediction model for predicting overall survival of esophageal cancer patients receiving neoadjuvant chemotherapy in the training cohort and the 1-year (**D**), 2-year (**E**) and 3-year (**F**) calibration curves in the validation cohort;.

**Figure 4:**
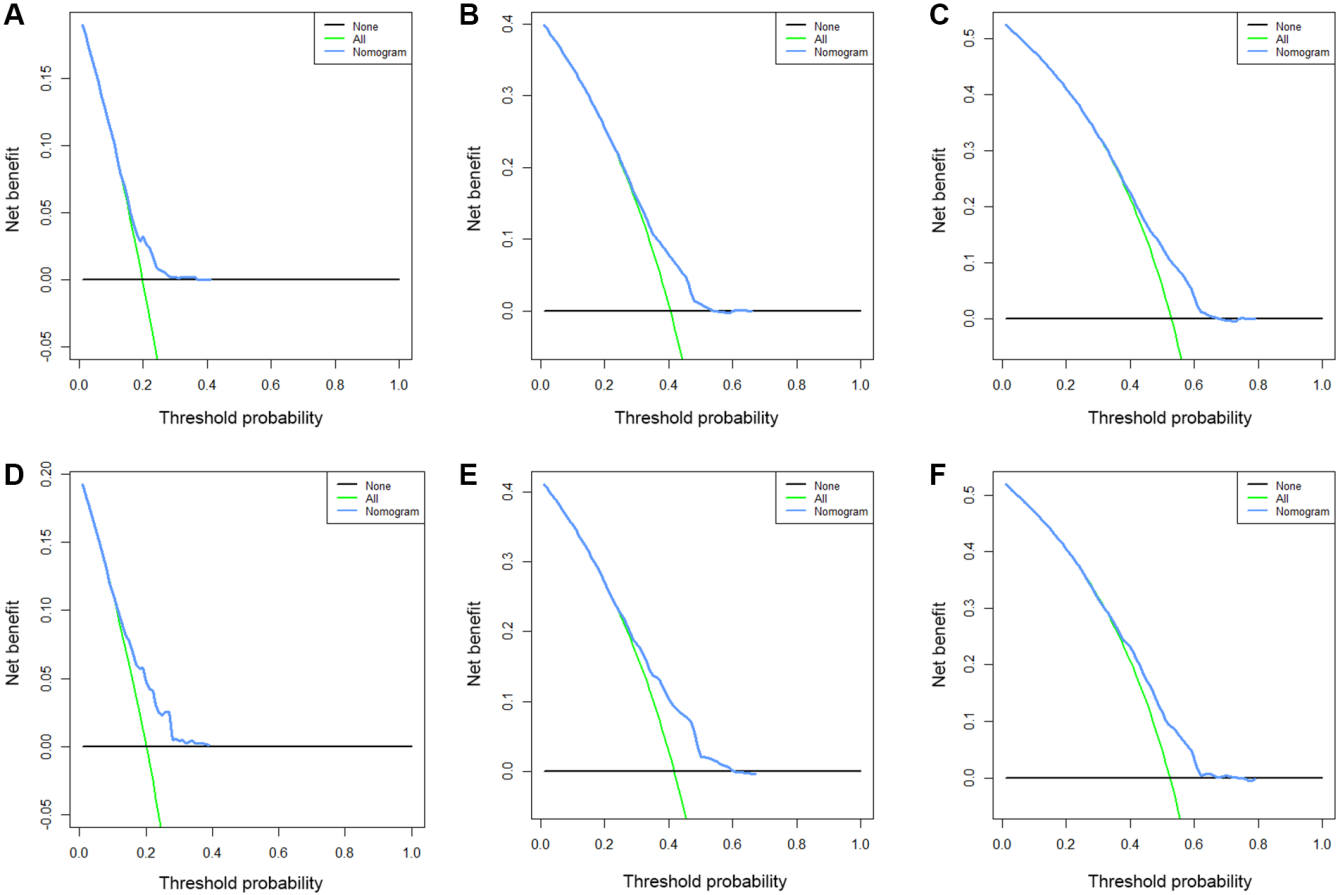
The 1-year (**A**), 2-year (**B**) and 3-year (**C**) DCA curves of the nomogram prediction model for predicting overall survival of esophageal cancer patients receiving neoadjuvant chemotherapy in the training cohort and the 1-year (**D**), 2-year (**E**) and 3-year (**F**) DCA curves in the validation cohort.

Based on the results of multivariate Cox regression, six prognostic factors independently associated with OS were included to construct the nomogram for predicting the 1-year, 2-year, and 3-year OS of EC patients receiving neoadjuvant chemotherapy (Figure 1). To facilitate the use of the model, we created an on-line nomogram (https://shubei11.shinyapps.io/nomogramforos/). In the nomogram model, the individual score of each variable could be obtained according to the variable situation of each patient, and the total score of the patient can be obtained by accumulating each individual score. A vertical line was drawn down from the total score to obtain the estimated OS at 1, 2, and 3 years for this patient. In the training cohort, the area under the ROC curve (AUC) of the 1-year, 2-year and 3-year OS nomogram were 0.598, 0.619 and 0.624, respectively, while in the validation cohort, the AUC of the 1-year, 2-year and 3-year OS nomogram were 0.632, 0.642 and 0.626, respectively (Figures 2A,B). In general, the constructed nomogram had moderate predictive ability. In addition, the time correlation ROC curve indicated that the establised nomogram constructed was better than the traditional TNM staging system in predicting OS at almost all time points (Figures 2C,D). Calibration curve analysis revealed a high degree of agreement between the 1-year, 2-year, and 3-year OS predicted by the nomogram and the actual prognostic outcomes in both the training cohort and the validation cohort(Figure 3). The results of DCA showed that the nomogram established in this study had excellent clinical practical application efficacy in predicting the 1-year, 2-year and 3-year OS of esophageal cancer patients receiving neoadjuvant chemotherapy (Figure 4).

### Risk stratification and Kaplan-Meier survival analysis based on nomogram score

**Figure 5:**
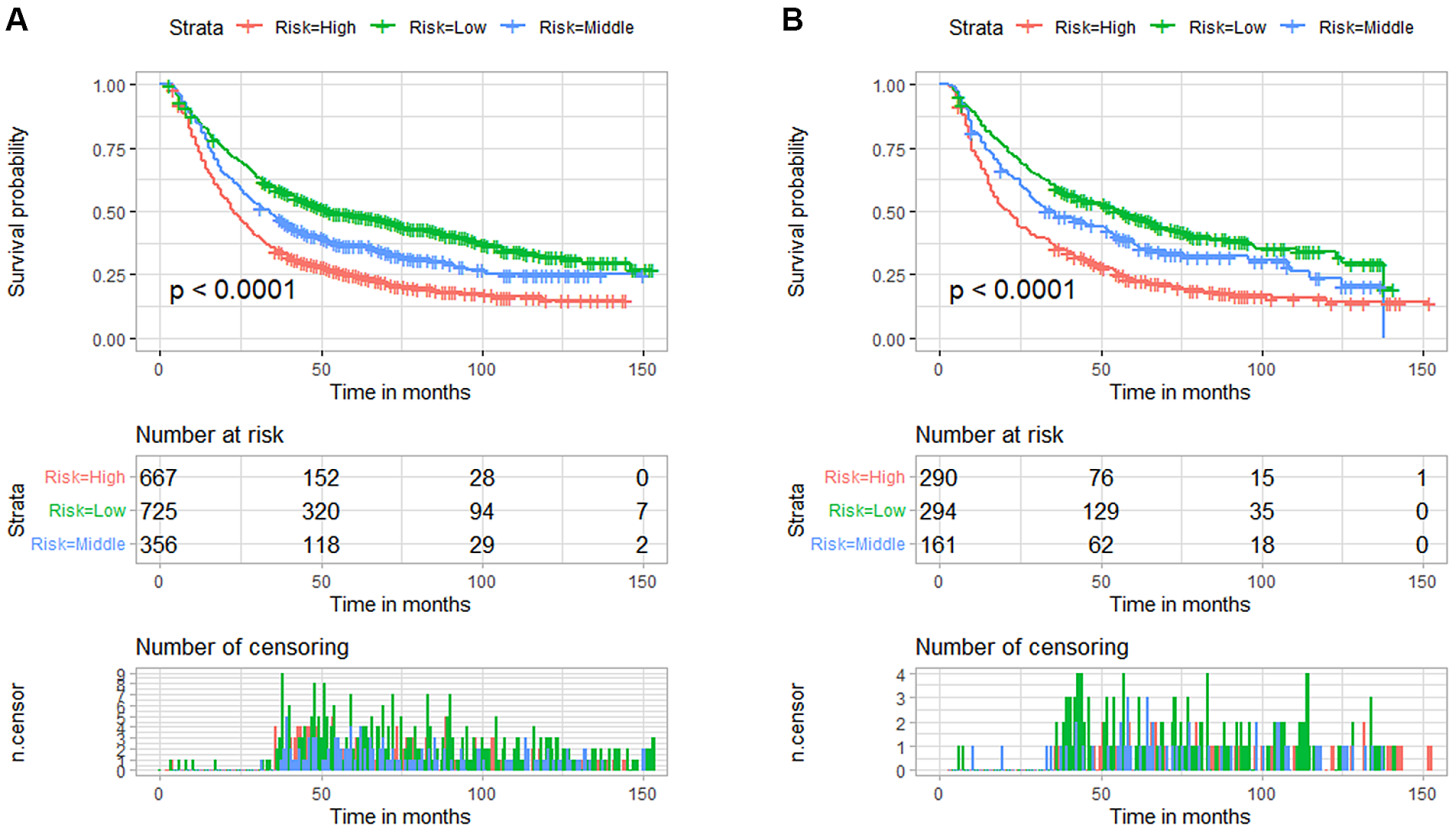
Kaplan-Meier survival curves for the three risk subgroups in the training cohort (**A**) and validation cohort (**B**) based on the nomogram prediction model for predicting overall survival in patients with esophageal cancer receiving neoadjuvant chemotherapy.

We divided the included patients into three risk subgroups according to the cut-off point analysis of X-Tile procedure, including the low-risk group (<174 points), the medium-risk group (174–192 points), and the high-risk group (>192 points). Then K-M survival analysis was performed, and the results showed that patients in the high-risk group always had a worse prognosis than those in the low-risk group in both the training and validation cohorts (Figure 5). The risk classification system based on nomogram had significant predictive value for the prognosis of EC patients receiving nCT.

## Discussion

With the promotion and application of nCT, the clinical management mode and OS of patients with EC are improved. In a randomized controlled trial conducted by Allum WH et al., the R0 excision rate, progression-free survival, and OS were significantly better in the nCT group than in the non-nCT group.The 5-year OS in nCT group and Non-nCT group were 23.00% and 17.10%, respectively (*P *= 0.003). Subgroup analysis showed that the 5-year overall survival rate of patients with ESCC (25.50% vs. 17.00%) and EAC (22.60% vs. 17.60%) in the nCT group were better than those in the non-nCT group. The efficacy of nCT is consistent in different histological types of EC (11). In addition, Ychou M et al. also concluded that receivingnCT is helpful to improve the radical resection rate, disease-free survival rate and OS of patients with EAC (12). Although the OS of patients with EC has been significantly improved by the development of nCT, the prognosis cann't be effectively evaluated by the present AJCC TNM staging system. In the prognostic studies of other common malignant tumors (13, 14), researchers have found that in addition to TNM stage, some other clinicopathological factors are also closely related to the prognosis. Moreover, more importantly, these studies have established prediction models for predicting the prognosis of cancer patients based on independent prognostic risk factors, and demonstrated that the established model is better than the AJCC staging system.It is not accurate to judge the prognosis of tumor patients only by AJCC TNM staging system, and even patients in the same staging may have significantly different survival times. More importantly, TNM staging system cannot meet the growing demand of precision medicine, nor can it provide individual prognosis prediction at a specific time (15, 16). Recently, nomogram prediction models that comprehensively consider various independent prognostic factors have been widely investigated and developed (17, 18). Nomogram is one kind of prediction model based on statistical method and risk score formula to graphically show the survival rate of patients at a specific time. By summing the corresponding scores of all independent prognostic factors, the predicted survival rate for the corresponding years can be obtained by drawing a straight line downward. More importantly, previous studies have shown that the integration of multivariate nomogram is better than single variable in predicting the prognosis of patients, showing higher prediction accuracy.

In this study, we included and analyzed the clinical data of 2,493 patients with EC who received nCT to construct a nomogram model to predict the OS. Six independent prognostic factors were identified by univariate and multivariate COX regression analysis, including T stage, N stage, M stage, pathological grade, marital status and gender. A novel nomogram model to predict 1-year, 2-year, and 3-year OS was established. We confirmed that the model has good discriminative power and clinical application ability. In addition, the newly developed prediction model is superior to the traditional TNM staging system in predicting the OS. To the best of our knowledge, this is the first study to construct a prognostic nomogram model for EC patients receiving nCT based on a large population. This nomogram can help identify high-risk subgroups that may require more intensive treatment. In addition, for high-risk subgroups in the entire population identified by this nomogram, we should pay close attention and shorten the follow-up interval, and treatment could be adjusted timely. We should also provide patients in high-risk with more psychological or emotional support, if necessary.

Consistent with TNM staging system (19–21), this study found that patients with higher T, N and M stages had worse prognosis. The prognosis of patients with larger tumor size is worse, which may be related to the difficulty of surgical resection of local invasion of tumor. In clinical practice, larger tumors often indicate that it is more difficult to completely remove the tumor and obtain an R0 resection margin. At the same time, large tumors are usually accompanied by abundant neovascularization, which greatly increases the risk of blood-borne metastasis due to extrusion during surgery (22). The presence of lymph node involvement and metastasis to distant organs often indicates that the patient's primary tumor is more aggressive. Many studies have suggested that male and female cancer patients have different survival rates, and in a nationwide cohort study of 23,465 participants with lung adenocarcinoma, female lung adenocarcinoma patients had slightly higher tumor-specific survival rates than male patients (23). Meanwhile, female patients with tumor-specific survival may benefit more from the use of platinum-based chemicals (24). Similar to the findings in previous studies, female had a better OS in EC patients receiving nCT. Shi et al. found that tumor pathologic stage is an early death of patients with stage IV esophageal independent risk factors (25). This study indicated that EC patients receiving nCT with higher pathologic stage had a worse prognosis. We contributed this finding to that the high undifferentiated tumor differentiation tumor often lead to a more invasive condition. In addition, one of the surprising findings of this study was that marital status was also significantly associated with the outcome of EC patients receiving nCT. Married patients had better long-term OS. Married patients have stronger financial resources and are more able to afford expensive treatment to achieve a better prognosis (26). On the contrary, due to the lack of support from family members, unmarried patients may have a tendency to experience financial difficulties and decreased ability to pay (27). However, they have to pay almost the same amount, and the economic burden of the disease increases accordingly for them. Thus, the marital status would affect the overall prognosis of tumor patients to a certain extent.

This study still has some unavoidable limitations in study design, clinical data collection, and validation. Firstly, this is a retrospective study based on the SEER database, and the absence of some clinical variables inevitably leads to data bias. Secondly, although the SEER database has the advantage of large study samples from database sources, it also has a series of limitations in terms of data collection. For example, there is a lack of routinely available clinical data, such as specific patient underlying performance status, comorbidities, and laboratory tests. Thirdly, the absence of molecular biological information and specific chemotherapy/chemoradiotherapy protocols is also a drawback of the SEER database. Finally, the established nomogram still lacks external validation of the predictive power of the model from different regional study cohorts.

## Conclusions

Gender, tumor pathological grade, marital status, T stage, N stage and M stage were identified as independent prognostic factors for overall survival of patients with esophageal cancer who received neoadjuvant chemotherapy. A nomogram prediction model was established, which was helpful to accurately and reliably predict the overall survival rate of patients with esophageal cancer who received neoadjuvant chemotherapy at 1, 2 and 3 years.

## Data Availability

The original contributions presented in the study are included in the article/Supplementary Material, further inquiries can be directed to the corresponding author/s.
